# Genetic Analysis of the Early Natural History of Epithelial Ovarian Carcinoma

**DOI:** 10.1371/journal.pone.0010358

**Published:** 2010-04-26

**Authors:** Bhavana Pothuri, Mario M. Leitao, Douglas A. Levine, Agnès Viale, Adam B. Olshen, Crispinita Arroyo, Faina Bogomolniy, Narciso Olvera, Oscar Lin, Robert A. Soslow, Mark E. Robson, Kenneth Offit, Richard R. Barakat, Jeff Boyd

**Affiliations:** 1 Department of Surgery, Memorial Sloan-Kettering Cancer Center, New York, New York, United States of America; 2 Molecular Biology Program, Memorial Sloan-Kettering Cancer Center, New York, New York, United States of America; 3 Department of Epidemiology and Biostatistics, Memorial Sloan-Kettering Cancer Center, New York, New York, United States of America; 4 Department of Pathology, Memorial Sloan-Kettering Cancer Center, New York, New York, United States of America; 5 Department of Medicine, Memorial Sloan-Kettering Cancer Center, New York, New York, United States of America; 6 Fox Chase Cancer Center, Philadelphia, Pennsylvania, United States of America; Health Canada, Canada

## Abstract

**Background:**

The high mortality rate associated with epithelial ovarian carcinoma (EOC) reflects diagnosis commonly at an advanced stage, but improved early detection is hindered by uncertainty as to the histologic origin and early natural history of this malignancy.

**Methodology/Principal Findings:**

Here we report combined molecular genetic and morphologic analyses of normal human ovarian tissues and early stage cancers, from both *BRCA* mutation carriers and the general population, indicating that EOCs frequently arise from dysplastic precursor lesions within epithelial inclusion cysts. In pathologically normal ovaries, molecular evidence of oncogenic stress was observed specifically within epithelial inclusion cysts. To further explore potential very early events in ovarian tumorigenesis, ovarian tissues from women not known to be at high risk for ovarian cancer were subjected to laser catapult microdissection and gene expression profiling. These studies revealed a quasi-neoplastic expression signature in benign ovarian cystic inclusion epithelium compared to surface epithelium, specifically with respect to genes affecting signal transduction, cell cycle control, and mitotic spindle formation. Consistent with this gene expression profile, a significantly higher cell proliferation index (increased cell proliferation and decreased apoptosis) was observed in histopathologically normal ovarian cystic compared to surface epithelium. Furthermore, aneuploidy was frequently identified in normal ovarian cystic epithelium but not in surface epithelium.

**Conclusions/Significance:**

Together, these data indicate that EOC frequently arises in ovarian cystic inclusions, is preceded by an identifiable dysplastic precursor lesion, and that increased cell proliferation, decreased apoptosis, and aneuploidy are likely to represent very early aberrations in ovarian tumorigenesis.

## Introduction

The high mortality to incidence ratio associated with epithelial ovarian carcinoma (EOC) reflects the fact that most of these cancers have spread beyond the ovary at the time of diagnosis [1]. The good prognosis for patients with disease confined to the ovary suggests that early detection could lessen mortality substantially. However, early detection is hindered by the lack of a proven screening modality. The use of serum CA-125 measurements and radiologic technology, alone or in combination, has yet to prove effective in either the high-risk or general populations [2], although the detection of diagnostic serum proteomic patterns or biomarker panels holds promise [3], [4]. Among the requirements for development of novel screening strategies for any cancer type are an understanding of the tumor's early natural history, including characterization of the histologic region of origin and a recognizable premalignant lesion, neither of which has been elucidated with respect to EOC.

There exist several theories on the pathogenesis of EOC [5]. While it is widely believed that the epithelial component of the ovary gives rise to the common epithelial ovarian carcinomas [6], it is not clear whether these cancers originate in the single-cell layer of surface epithelium or in architectural aberrations of the surface epithelium. These include surface epithelial-lined clefts and cortical inclusion cysts, thought to result from post-ovulatory wound repair, tissue remodeling associated with pregnancy or aging, paraovarian adhesions, or simply the dynamic interaction between surface epithelium and underlying stroma [7]–[9]. A related debate centers on whether these morphologic alterations of surface epithelium are more prevalent in the ovaries of women who have developed EOC or are at high genetic risk for EOC, and there are conflicting data with respect to both issues. This is perhaps not surprising, since from a statistical power perspective, it would be very difficult to adequately address either hypothesis.

An alternative histopathology-based theory holds that EOC may arise in components of the secondary Müllerian system, located within or adjacent to the ovary [10]. This theory is supported by the high likelihood that at least some proportion of EOCs of endometrioid or clear cell histologic types arise from endometriotic lesions of the ovary [6]. In addition, the ovarian surface epithelium is actually a modified mesothelium, contiguous with and morphologically resembling the peritoneal mesothelial lining. Typical EOCs, however, are readily distinguished from the very rare primary ovarian mesothelioma, and instead resemble carcinomas arising in true Müllerian-derived tissues such as the endocervix, endometrium, and fallopian tube, suggesting the requirement for a metaplastic process if EOCs do in fact arise from the ovarian surface mesothelium.

Similarly, in an even more radical departure from traditional models of ovarian tumorigenesis, an emerging theory suggests that type II (high grade serous) EOCs are perhaps not ovarian cancers at all, but rather originate in the fallopian tube [11]. This hypothesis, first developed by Piek and colleagues [12], was based on the observation of dysplastic morphologic and preneoplastic molecular alterations in prophylactically removed fallopian tubes from women with germline *BRCA* mutations. Subsequently, several research groups have reported on the striking prevalence of histopathologic and/or molecular genetic alterations observed in fallopian tubes from women with *BRCA* mutations who have undergone risk-reducing or ovarian cancer surgery. This body of clinicopathologic and molecular evidence warrants serious consideration of this model as it may apply to at least some proportion of type II EOCs.

The existence of an identifiable premalignant lesion for EOC is also debated, a problem compounded by the uncertainty regarding its histologic origin, and the fact that most EOCs are of advanced stage and associated with little or no evidence of preinvasive or normal epithelium at the time of pathological diagnosis. Candidate precursor lesions include dysplasia [13], hyperplasia [14], and more subtle alterations involving cellular or nuclear “atypia” [15], [16]. An argument for the development of ovarian carcinoma “de novo”, in the absence of any intermediate precursor lesion, has also been presented [17].

The advent of new molecular biological and genetic information coupled with powerful technologies has begun to allow the study of this problem at a level beyond that of the purely morphologic, as in the observation of loss of heterozygosity at the *BRCA1* and *TP53* loci in an ovarian “carcinoma in situ” lesion from a *BRCA1* mutation carrier [18]. Here we combine cellular morphologic and molecular genetic analyses to address the hypothesis that EOC may arise in ovarian cystic inclusions.

## Results

### Analysis of Ovarian Tissues from Genetically High Risk Individuals

Using a combined genetic and morphologic approach, we first sought evidence that early ovarian carcinoma histogenesis could be observed in ovarian tissues from *BRCA* heterozygotes, who have a 20–40% lifetime risk of ovarian cancer [19]. Two somatic molecular genetic alterations are minimally required for cancer development in this population, loss of the wild-type *BRCA* allele and, in a majority of cases, mutational inactivation of the *TP53* gene [20], both of which may be used to search for preclinical genetic evidence of ovarian tumorigenesis.

**Figure 1:**
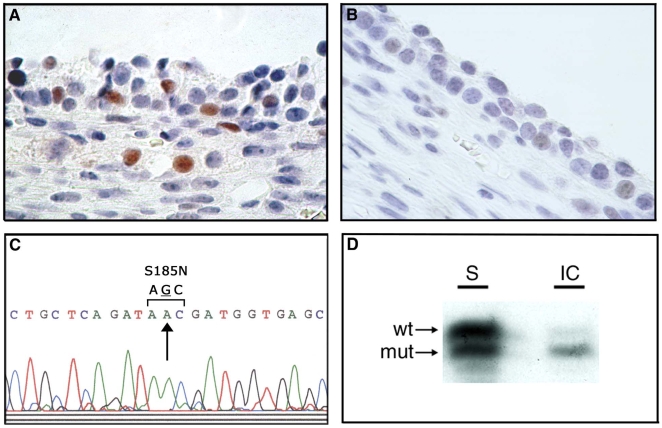
Assessment of p53/*TP53* status and *BRCA2* allelotype in epithelial inclusion cysts of normal ovary from a *BRCA2* 6174delT heterozygote (specimen PO67 in Supplemental Table S1). (A) p53 immunopositive cells from an epithelial inclusion cyst. (B) p53 immunonegative cells from a different epithelial inclusion cyst from the same ovary. (C) Sequence analysis of *TP53* in p53 immunopositive cells from inclusion cyst shown in panel (A). A missense mutation is evident at codon 185 (AGC→AAC; S185N). (D) Loss of the wild-type (wt) *BRCA2* allele in DNA derived from the inclusion cyst (IC) shown in panel (A), with retention of mutant (mut) and wild-type alleles in DNA derived from surface (S) epithelium from the same ovary.

Immunohistochemical analysis of p53 expression was used to assess the presence of generalized genetic damage and/or *TP53* mutation in 37 pathologically normal prophylactic oophorectomy specimens from *BRCA* heterozygotes. Focal nuclear p53 expression was observed in 10 (27%) specimens, invariably confined to morphologic alterations of the epithelium such as cortical clefts and inclusion cysts (Figure 1A). Expression of p53 was not observed in surface epithelial cells or in nonepithelial components of the ovary, and expression was usually confined to a single focus of epithelium in each ovary (Figure 1B). To perform a genetic analysis of these cells, laser catapult microdissection of p53-immunopositive and immunonegative cells from each ovary was accomplished, followed by DNA isolation from the pooled cells from each aberrant histologic focus. Sequence analysis of *TP53* revealed a mutation in three p53-immunopositive specimens (Figure 1C), two from *BRCA1* heterozygotes and one from a *BRCA2* heterozygote. All were missense mutations occurring in exons 5 or 6 (Supplemental Table S1). No mutations were detected in p53 immunonegative epithelial cells from the same ovaries.

Next, genetic evidence for loss of the wild-type *BRCA* allele was sought in the 10 p53-immunopositive tissue specimens. Epithelial cells containing the *TP53* mutation in specimens PO3 and PO67 also displayed loss of heterozygosity (LOH) of the wild-type *BRCA1* or *BRCA2* alleles, respectively (Figure 1D; Supplemental Table S1). No evidence of *BRCA* LOH was observed in the cells from PO49, which contained a *TP53* mutation, or in the seven additional specimens in which p53 overexpression, but not mutation, was observed. These data indicate that *BRCA* LOH and *TP53* mutation may coexist in epithelial inclusions prior to the appearance of a pathological lesion in ovaries from *BRCA* heterozygotes, and that *TP53* mutation may precede *BRCA* LOH in some cases. The frequent overexpression of p53 in the absence of *TP53* mutation in epithelial inclusion cysts of *BRCA* heterozygotes suggests that genetic damage, aberrant growth signals, and/or other types of oncogenic cellular stress [21] are common in this histologic region of ovaries from these individuals.

**Figure 2:**
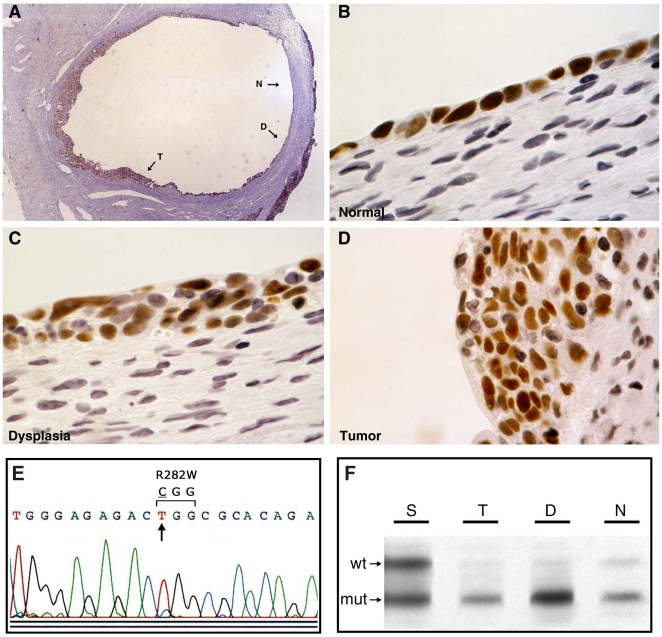
Morphological, immunohistochemical, and genetic analyses of early stage ovarian carcinoma arising in a *BRCA1* 185delAG heterozygote (specimen OC16 in Supplementary Table S2). (A) Low-power photomicrograph of histologic progression of normal epithelium (N) to dysplastic epithelium (D) to invasive carcinoma (T) arising within an inclusion cyst. (B,C,D) High-power photpmicrographs of cellular regions of normal, dysplastic, and carcinoma, respectively, as shown in panel (A). The immunostain is for p53 in panels (A–D). (E) Sequence analysis of *TP53* representative of DNA samples from all three cellular components shown in panels (B–D). A missense mutation is evident at codon 282 (CGG→TGG; R282W). (F) Loss of the wild-type (wt) *BRCA1* allele in DNA derived from normal, dysplastic, and tumor cells shown in panels (B–D), with retention of mutant (mut) and wild-type alleles in surface epithelium from the same ovary.

To determine whether these early genetic and morphologic alterations in clinically normal ovaries were likely to be relevant to ovarian tumorigenesis, we identified five cases of early stage ovarian carcinoma from confirmed *BRCA* heterozygotes (Supplemental Table S2). In all five cases, the carcinoma was found to arise in a morphologic alteration of the surface epithelium, an inclusion cyst in four cases and a surface papillation in the one case (OC15). Furthermore, a histologic continuum of normal epithelium, dysplasia, and carcinoma was evident within the foci of epithelial aberrations in all five cases. (We defined dysplasia according to general histopathological principles related to alterations in size, shape, and organization of the cellular components of a tissue. Specifically, the four criteria used in this study were: 1) cellular pleomorphism; 2) nuclear atypia; 3) loss of cellular architectural organization; and 4) absence of stromal invasion.) These cases were subjected to immunohistochemical analysis of p53 expression, with strong nuclear overexpression observed in three of the five carcinomas (OC6, OC7, and OC16). In the p53-immunopositive cases, similar nuclear overexpression was observed in the adjacent dysplastic and normal epithelium for all three cases (Figure 2A-D; Supplemental Figures S1 and S2). Laser catapult microdissection and DNA isolation from the normal epithelium, dysplasia, and carcinoma components of each case were performed, and genetic analysis of *TP53* status revealed a missense mutation in all three p53-immunopositive cases (Figure 2E; Supplemental Table S2). Additionally, the two p53-immunonegative tumors were found to harbor frameshift mutations in *TP53* (Supplemental Table S2). Genetic analyses of DNA from the normal epithelium and dysplasia adjacent to the carcinomas revealed the presence of the corresponding tumor-associated *TP53* mutation in normal epithelium in two of five cases, and in dysplasia but not normal epithelium in two additional cases (Supplemental Table S2).

The various histologic components of each cancer case were then subjected to genetic analysis for loss of the wild-type *BRCA* allele. As expected, *BRCA* LOH was observed in the invasive cancer components from all five cases. Analysis of the dysplasia and normal epithelium adjacent to the carcinomas revealed *BRCA* LOH in normal epithelium and dysplasia in two cases, and in dysplasia but not normal epithelium in one additional case (Figure 2F; Supplemental Table S2). Either *TP53* mutation or *BRCA* LOH was evident in dysplastic cells from all evaluable cases. From a molecular genetic perspective, these data support the inference drawn from analysis of the prophylactic oophorectomy specimens, that loss of the wild-type *BRCA* allele may precede or follow *TP53* mutation during the early stages of *BRCA*-associated ovarian tumorigenesis, but that in either case, these genetic events are closely linked. Both of these molecular genetic scenarios are consistent with the cell “death by checkpoint” model of BRCA function [22].

### Relevance of Model to Sporadic Ovarian Tumorigenesis

These findings support a model of *BRCA*-linked ovarian carcinoma histogenesis in which clonal progression of normal epithelium through a dysplastic precursor lesion to invasive carcinoma occurs within epithelial inclusion cysts. We next determined whether this model is applicable to the more common sporadic manifestation of ovarian carcinoma. Expression of p53 was assessed in 20 pathologically normal ovaries removed for benign indications from unselected patients not known to be at high risk for ovarian cancer. Nuclear p53 expression was apparent in five (25%) of these cases; immunopositive epithelial cells were localized to inclusion cysts in four cases and within a cortical invagination in one case. Expression of p53 was not observed in surface epithelial cells (Supplemental Figure S3). Sequence analysis of *TP53* using DNA from microdissected immunopositive cells failed to reveal a mutation in any case. These findings again suggested that the epithelial cells within ovarian inclusion cysts are particularly susceptible to oncogenic cell stress, leading to p53 accumulation, but did not provide clear evidence of neoplastic progression as observed in normal ovarian tissues from women with *BRCA* mutations. Others have noted the expression of p53 in ovarian inclusion cysts or deep invaginations in the presence of cellular “atypia” [23] or “hyperplasia/dysplasia” in prophylactic oophorectomy specimens [24].

To search further for evidence of multistep neoplastic progression in ovarian tissues, we examined a consecutive series of 145 stage I/II ovarian cancers, also from women not known to be at high genetic risk for ovarian cancer, and from this series identified 23 cases in which an ovary had retained any evidence of an epithelial component in addition to the carcinoma. The carcinoma was found to arise in an inclusion cyst in 21 (91%) of these cases, in a cortical invagination in one (4%) case, and from the surface epithelium in one (4%) case. In all 23 cases, the noninvasive epithelial component consisted of normal epithelium and dysplasia directly adjacent to the carcinoma (Supplemental Figures S4 and S5).

To determine the relationship of carcinoma to the adjacent dysplastic and normal epithelial cells within these lesions, assessment of *TP53*/p53 status was performed as before. The normal and dysplastic epithelium adjacent to cancers contained p53 immunopositive cells in 12 cases (52%) (Supplemental Table S3; Supplemental Figures S4 and S5); other epithelial cells and other cell types in the same ovaries were invariably immunonegative for p53. These data again indicate the presence of oncogenic cell stress and p53 upregulation and/or mutation in normal epithelial and dysplastic cells adjacent to invasive cancers within inclusion cysts, but not elsewhere within the same ovarian tissues.

Sequence analysis of *TP53* was performed using DNA from microdissected carcinoma cells from each case. Mutations were identified 11 of 23 (48%) invasive cancers (Supplemental Table S3). In two of these cases, the same *TP53* mutation was present in the adjacent dysplastic and normal epithelial components, confirming their precursor relationship with the associated invasive carcinoma. In the other nine cases, however, no evidence of the *TP53* mutation was observed in either normal epithelial or dysplastic cells, implying that in most cases of sporadic ovarian carcinoma in which *TP53* mutation occurs, the mutation is not detectable until the transition of dysplasia to carcinoma.

### Gene Expression Profiles of Ovarian Surface and Cystic Epithelial Cells

To gain insight into the cellular factors that create an environment compatible with enhanced DNA damage and oncogenesis within ovarian epithelial inclusion cysts, we performed gene expression profiling of microdissected epithelial cells from ovarian cystic inclusions compared to ovarian surface epithelial cells from the same pathologically normal ovaries. Differential expression of 1,443 genes was observed between cystic and surface epithelial cells (Supplemental Table S4), suggesting the presence of distinct gene expression phenotypes in the two cell populations.

To test whether the cystic epithelial cell expression profile was more similar to that of ovarian carcinoma than that of the surface epithelial cells, a three-way comparison was made incorporating RNA from ovarian cancers. Differential expression of 657 genes in cystic epithelium and cancer compared to surface epithelium (418 up-regulated and 239 down-regulated; Supplemental Table S5) was observed, compared to only 276 genes differentially expressed in surface epithelium and cancer compared to cystic epithelium (88 up-regulated and 188 down-regulated; Supplemental Table S6). Notably, however, functional annotation of the differentially expressed genes (Supplemental Table S5) indicated the presence of a quasi-neoplastic molecular phenotype in the ovarian cystic epithelium, with up-regulation of oncogenic factors (e.g., *EZH2*, *EVI1*, *MYB*, *ERBB4*, *CCNE1*, *MEIS1*, *KRAS2*, *MMP7*, *VEGF*, *MAPK1*, *MAPK9*) and cancer-specific antigens (e.g., *TACSTD1*, *WFDC2*, *CD44*, *CD24*), and down-regulation of tumor suppressors (e.g., *GADD45B*, *GAS1*, *PTEN*, *BAX*). This degree of co-expression of cancer-related genes was not evident in the surface epithelial cell/tumor vs. cystic epithelial comparison (Supplemental Table S6).

**Figure 3:**
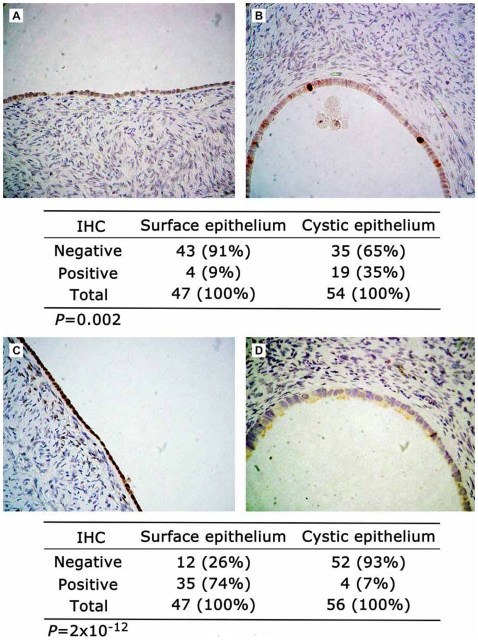
Semiquantitative immunohistochemical analyses of gene products differentially expressed in normal ovarian cystic epithelium and ovarian carcinoma compared to normal ovarian surface epithelium. (A) Normal ovarian surface epithelium displaying negative immunstaining for TOP2A. (B) Normal ovarian cystic epithelium displaying strong immunopositivity for TOP2A in two cells. (C) Normal ovarian surface epithelium displaying immunopositivity for FOS in all cells. (D) Normal ovarian cystic epithelium displaying negative immunostaining for FOS. Indicated under each pair of photomicrographs are the proportions of immunopositive and immunonegative cellular compartments for the indicated number of ovarian specimens, with the associated *P*-values.

To estimate the quality of the expression profiling data, we performed immunohistochemical analyses of expression for two arbitrarily chosen gene products, one overexpressed in cyst epithelium and tumor compared to surface epithelium (TOP2A) and one underexpressed in cyst and tumor compared to surface epithelium (FOS), using 56 pathologically normal ovaries (Figure 3). Semi-quantitative analyses of protein expression revealed highly statistically significant differences between the two groups in accordance with the microarray-based data. We then compared the list of genes differentially expressed in cyst epithelium and tumor compared to surface epithelium to gene lists generated in 11 previous studies reporting expression profiles of ovarian carcinomas compared to one or another normal control (data not shown). Seventy-one of the genes listed on Supplemental Table S4 had been previously noted to be differentially expressed in at least one of these reports, and two genes *TACSTD1* (encoding tumor-associated calcium signal transducer 1) and *WFDC2* (encoding whey acidic protein type, four-disulfide core domain 2, or putative ovarian carcinoma marker HE4) had been noted in five and six previous reports, respectively, suggesting that overexpression of these genes may play critical roles in the early stages of ovarian tumorigenesis.

To extract potentially meaningful biological insights form these hypothesis-generating gene expression profiling studies, we employed the EASE (Expression Analysis Systematic Explorer) theme discovery tool [25] to search for evidence of specific molecular biological pathways that may be disrupted in cystic ovarian epithelium. Using this approach, three categories of genes were identified (Supplemental Table S7), including down-regulation of those with signal transduction activity, up-regulation of those involved in mitotic cell cycle control, and up-regulation of those involved in microtubule organization and biogenesis. Notably, forced overexpression of several of the genes from the third category, including MAP7 [26], [27], PCNT2 [28], [29], TBCD [30], PRC1 [31] leads to defective mitotic spindle assembly. From this analysis, we hypothesized that the extracellular environment in the ovarian epithelial inclusion cyst causes gene expression changes leading to attenuation of specific signal transduction pathways, inappropriate cell cycle progression, and defective mitotic spindle assembly, resulting in aneuploidy. The latter two components of this hypothesis could be readily tested.

### Cell Proliferation Index and Ploidy

To quantitate the cell proliferation index in epithelial inclusion cysts and surface epithelial cells of the ovary, we examined an independent set of 36 pathologically normal ovaries. Cell proliferation was significantly higher in cyst compared to surface epithelial cells, and apoptosis was significantly higher in surface compared to cystic epithelial cells (Tables 1 and 2, and Supplemental Figure S6). The cell proliferation index (ratio of cell proliferation to apoptosis) was nine-fold higher in epithelial cells of inclusion cysts compared to surface epithelial cells of the normal ovary.

**Table 1:** Cell proliferation indices in ovarian cystic vs. surface epithelial cells.

Tissue/CellType	No. Samples	No. Nuclei	No. Positive Nuclei (%)	*P*
**Cell Proliferation**				
Ovaries	36	39,382	220 (0.55%)	
Surface Epithelial	28	19,216	64 (0.33%)	
Cystic Epithelial	32^a^	20,616	156 (0.76%)	0.04
**Apoptosis**				
Ovaries	28	16,710	1,344 (8.0%)	
Surface Epithelial	20	5,544	863 (16%)	
Cystic Epithelial	24^b^	11,166	481 (4.3%)	<0.001

The “No. Samples” represents the number of independent ovarian tissues analyzed for each cell type. The tissues analyzed for apoptosis were a subset of those analyzed for cell proliferation. *P*-values apply to the comparison of values for cystic vs. surface epithelial cells.

a Contained 159 inclusion cysts.

b Contained 125 inclusion cysts.

**Table 2:** Cell proliferation index (CPI).

	Cell Proliferation	Apoptosis	CPI
Surface Epithelial	0.33%	16%	0.02
Cystic Epithelial	0.76%	4.3%	0.18
Ratio of Cyst CPI to Surface CPI			9

The CPI represents the ratio of cell proliferation to apoptosis for each cell type.

**Figure 4:**
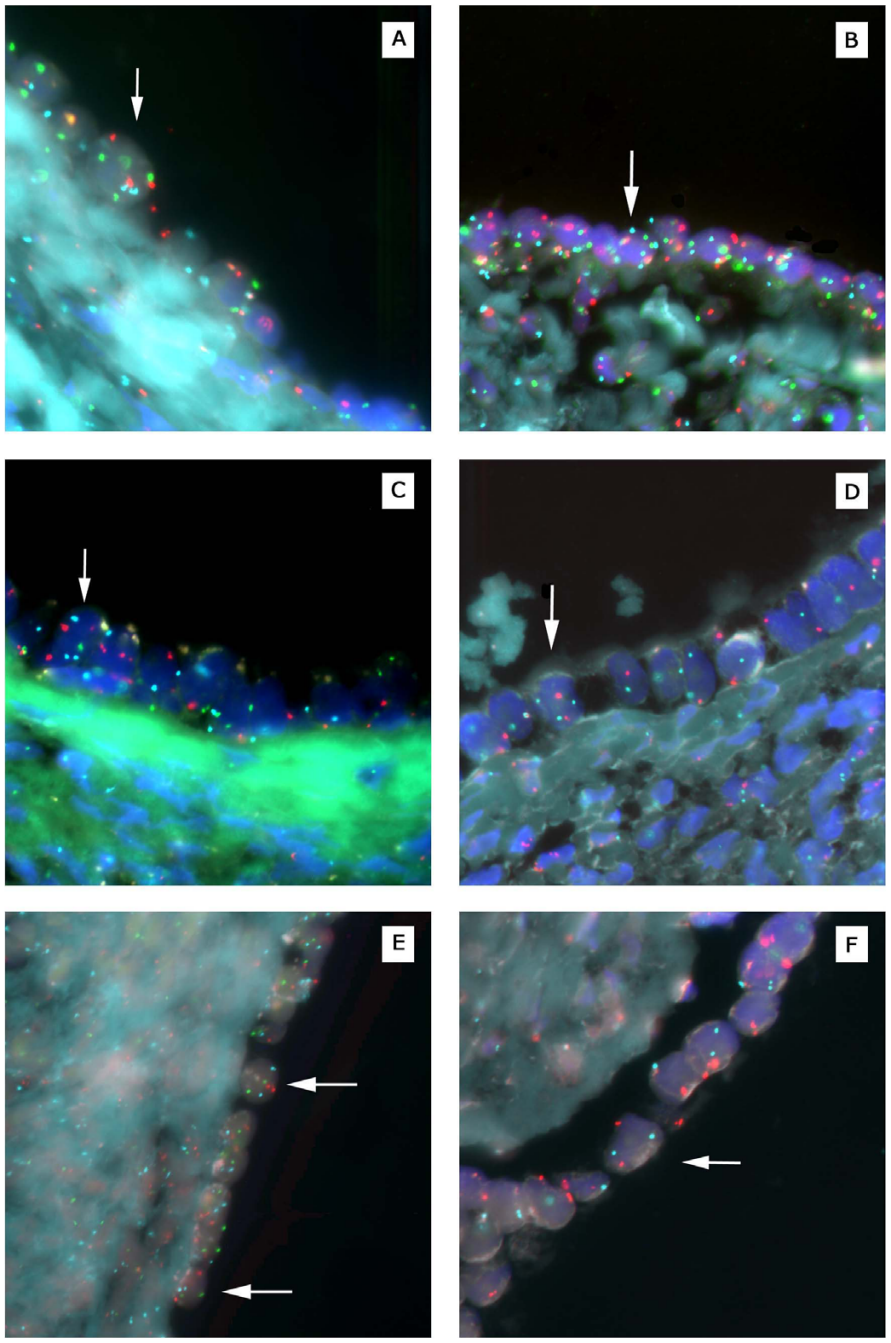
Fluorescence in situ hybridization analysis of ploidy in normal ovarian cystic and surface epithelia. All examples shown are from distinct ovarian specimens. (A) Cystic epithelial cell (arrow) from sample #112 (see Table 2) with three copies of chromosomes 3 (red) and 11 (green). (B) Cystic epithelial cell from sample #84 with three copies of chromosomes 6 (blue) and 11 (green). (C) Cystic epithelial cell from sample #135 with three copies of chromosome 6 (blue). (D) Cystic epithelial cell from sample #59 with three copies of chromosome 8 (blue). (E) Surface epithelial cells from sample # 13 with two copies of chromosomes 3 (red), 6 (blue), and 11 (green). (F) Surface epithelial cell from sample #102 with two copies of chromosomes 3 (red) and 8 (blue).

With respect to DNA content, ovarian surface epithelial cells were completely within the range for diploid control cells, whereas approximately 17% of the cystic epithelial cells displayed a DNA content that overlapped with the aneuploid control cell line (Supplemental Figure S7). The finding of a greater than diploid DNA content in cystic epithelial cells suggested the presence of aneuploidy. A portion of the same tissue specimens (*n* = 11) was then subjected to fluorescence in situ hybridization (FISH) analysis using centromeric probes for six chromosomes (1, 3, 6, 7, 8, 11). As shown in Figure 4 and Table 3, aneuploidy was evident in 9.2% of cystic epithelial nuclei but in only 0.5% of surface epithelial nuclei examined. Thus, aneuploidy was found to be relatively common in histopathologically normal epithelial cells from cystic inclusions, but very rare in surface epithelial cells.

**Table 3:** Cell ploidy as determined by fluorescence in situ hybridization.

Sample No.	Cellular Component	Triploid (%)	Tetraploid (%)	No. Probes
13	Surface (*n* = 47)	0	0	6
	Cyst (*n* = 85)	9 (10.6)	4 (4.7)	5
37	Cyst (*n* = 44)	14 (31.8)	1 (2.3)	4
59	Surface (*n* = 9)	0	0	3
	Cyst (*n* = 161)	14 (8.7)	2 (1.2)	6
84	Surface (*n* = 45)	0	0	6
	Cyst (*n* = 135)	5 (3.7)	0	6
86	Surface (*n* = 56)	1 (1.8)	0	3
	Cyst (*n* = 36)	1 (2.8)	0	5
102	Surface (*n* = 96)	0	0	4
	Cyst (*n* = 43)	2 (4.7)	0	3
112	Surface (*n* = 11)	0	0	3
	Cyst (*n* = 139)	10 (7.2)	2 (1.4)	6
131	Surface (*n* = 36)	1 (2.8)	0	5
	Cyst (*n* = 46)	6 (13.0)	2 (4.3)	5
135	Surface (*n* = 40)	0	0	5
	Cyst (*n* = 90)	4 (4.4)	0	6
140	Surface (*n* = 29)	0	0	3
	Cyst (*n* = 34)	1 (2.9)	0	3
183	Surface (*n* = 41)	0	0	4
	Cyst (*n* = 67)	4 (6.0)	0	5
**Total Ovaries**	**Total Cell Type**	**Total Triploid (%)**	**Total Tetraploid (%)**	**Total % Aneuploid**
11	Surface (*n* = 410)	2 (0.5)	0	0.5
	Cyst (*n* = 880)	70 (7.9)	11 (1.3)	9.2

The number of surface epithelial cells and number of cystic epithelial cells available for quantitation for each ovarian specimen are listed, followed by the number (percentage) of nuclei showing triploidy or tetraploidy for at least one of the indicated number of probes. Statistical analysis of these data is described in the Materials and Methods section; the difference between total percentage of aneuploid cells in cyst vs. surface epithelial cells is significant at a value of *P*<0.001.

## Discussion

The data support a model in which ovarian cancers frequently arise within epithelial inclusion cysts, but not the surface epithelium *per se*, and that carcinoma may be preceded by a dysplastic precursor lesion. A substantial degree of oncogenic stress exists within these epithelial inclusions, as evidenced by the frequent accumulation of p53. Inclusion cysts display a quasi-neoplastic expression signature; functional annotation reveals that pathways involving signal transduction, cell cycle progression, and mitotic spindle formation are significantly affected. These hypotheses were validated in experiments demonstrating that the cell proliferation index was markedly increased in cystic compared to surface epithelial cells, and that aneuploidy was present in normal-appearing cystic epithelial cells but not surface epithelial cells.

Allelic imbalance, a surrogate for aneuploidy, has been observed in small colonic adenomas, the earliest identifiable precursor lesion of colorectal carcinoma [32]. However, the finding of aneuploidy in histopathologically normal cells from the likely histologic region of origin of ovarian cancer suggests that this type of chromosomal instability is among the earliest of genetic events in the natural history of ovarian tumorigenesis, and to our knowledge, has no documented precedent in other human cancer types. It is important to note in this context that the large majority of ovarian carcinomas are aneuploid [33], [34]. The possibility that aneuploidy may occur as an early, initiating event and thus a driving force of tumorigenesis has been argued from the perspective of a mathematical genetic model [35].

These findings have elements in common with a model that expands on the concept of cell “death by checkpoint” [22], in which early human precancerous lesions are characterized by oncogenic stress (of an undefined nature), causing deregulated DNA replication and genomic instability, which in turn activates a DNA damage response that ostensibly delays or prevents cancer [36], [37]. In this model, a strong selective pressure then exists for mutations that compromise this checkpoint, for example in the ATM-Chk2-p53 pathway, resulting in tumorigenic progression. Our data prompt the hypothesis that the unique gene expression signature observed in ovarian epithelial inclusion cysts represents the “oncogenic stress” of this model, which in the ovarian inclusion cyst, leads to aberrant growth signals and DNA damage in the form of aneuploidy, both of which can cause p53 accumulation. The high prevalence of *TP53* mutations in hereditary [20] and sporadic [38] ovarian carcinomas suggests that this gene is a frequent mutational target of the selective pressure for aneuploid cells to overcome this checkpoint. Indeed, recent data support this concept that proliferation of aneuploid human cells is limited by a p53-dependent mechanism [39].

The absence of detectable *TP53* mutation in pathologically normal inclusion cysts that contain aneuploid cells provides evidence from human tissue *in vivo* that is consistent with the previous observation *in vitro* that inactivation of p53 does not, in and of itself, lead to the development of aneuploidy [40]. Although we have no evidence that genetic mutation of one or more “cancer CIN (chromosomal instability)” genes [41] has not occurred in aneuploid cystic epithelial cells, leading to the development of aneuploidy, our data are not inconsistent with a model of widespread epigenetic alterations in the expression of CIN genes, so designated because reconstitution of any of several of the epigenetic alterations observed in cystic inclusions confers CIN on a diploid cell in tissue culture, leading to the self-propagation of aneuploidy in the absence of specific mutations [26]–[31]. The concept of epigenetic events playing an important role in the development of aneuploidy has been proposed by others [42], [43].

Based on the model developed here, mutation of *TP53* would represent one requirement for the progression of cystic aneuploid cells to dysplasia and carcinoma. However, our data indicate that *TP53* mutation, found in most ovarian carcinomas, usually becomes detectable in the transition of dysplasia to carcinoma in sporadic tumors, although earlier in *BRCA*-linked hereditary tumors. It was not technically feasible to rule out the possibility that *TP53* is mutated in a small fraction of isolated cells in cystic inclusions, presumably among those that have developed aneuploidy, and that this mutational event occurs much earlier in tumorigenic progression. The observation of two sporadic stage I cancers and two *BRCA*-linked stage I cancers with the same *TP53* mutation detectable in normal, dysplastic, and carcinoma cells supports this hypothesis. It may be that clonal expansion of a cell(s) with a specific *TP53* mutation is often a late event (i.e., dysplasia to carcinoma), while isolated aneuploid cells in earlier precursor cell populations sustain distinct *TP53* mutations in a majority of those cells that progress toward cancer. The genes differentially expressed in ovarian inclusion cysts and cancers compared to surface epithelium may provide many candidates for mutated or aberrantly regulated oncogenes and tumor suppressor genes, markers for the early detection of ovarian cancer, and clues to the factors that induce oncogenic stress (i.e., a quasi-neoplastic gene expression profile) within ovarian cystic inclusions. Knowledge of the molecular basis for the induction of oncogenic stress characteristic of inclusion cysts could in turn lead to the development of novel ovarian cancer prevention strategies.

## Materials and Methods

### Ethics Statement

All human tissues used in this study were obtained and analyzed in accordance with a protocol approved by the Institutional Review Board of the Memorial Sloan-Kettering Cancer Center. Written informed consent was obtained from all participants in this study.

### Immunohistochemistry and TUNEL Assay

For assessment of gene expression by immunohistochemistry, 5 µm sections were prepared from archival, formalin-fixed, paraffin-embedded tissue specimens and processed using standard techniques. For p53, a mouse monoclonal antibody (clone DO-7, Dako) was used at a dilution of 1∶500. For FOS, purified rabbit polyclonal antiserum (SC52, Santa Cruz Biotechnology) was used at a dilution of 1∶400. For TOP2A, a mouse monoclonal antibody (clone SWT3D1, Oncogene Research Products) was used at a dilution of 1∶300. For Ki-67, a mouse monoclonal antibody (MIB1, Dakocytomation) was used at a dilution of 1∶75. Following the application of appropriate secondary antibodies, a standard streptavidin-biotin technique, with diaminobenzidine as chromogen, was used for visualization. The TUNEL assay was performed using the In Situ Cell Death Detection Kit (Roche Applied Science) according to the manufacturer's instructions. All slides were counterstained with hematoxylin, except those for the Ki-67 assay, which were counterstained with methyl green.

### Laser Catapult Microdissection

For isolating individual cells or clusters of cells for genetic analyses, the PALM Microbeam System (P.A.L.M. Microlaser Technologies AG) was used. This instrument employs a pulsed, low-energy 337 nm nitrogen laser coupled to an Axiovert 200 inverted microscope (Carl Zeiss). Groups of cells to be collected were localized and marked under the microscope and then isolated by laser microbeam microdissection, which forms a clear gap around the selected area. Laser-mediated catapulting of the selected cells resulted in their transfer into the cap of a 0.5 ml PCR reaction tube (PALM), autoclaved and DEPC-treated before use. The caps were then placed on tubes containing a digestion mixture consisting of 10 µg of PCR-grade proteinase K (Roche) in 50 µl of digestion buffer containing 10 mM Tris-HCl, pH 8.0, 1 mM EDTA, pH 8.0, and 1% Tween 20. The cells were suspended in the digestion mixture by microcentrifugation at 14,000 rpm for 2 min. Digestion was accomplished at 37°C for 16 hr, followed by 60°C for 2 hr. The proteinase K was then inactivated by heating to 95°C for 10 min, and samples were cooled to 4°C for storage. Prior to PCR-based genetic analyses, DNA was isolated and concentrated to a volume of 6 µl using the DNA Clean and Concentrator Kit (Zymo Research).

For isolation of cells from formalin-fixed, paraffin-embedded tissues, blocks were cooled on ice for 20 min, and 8 µm sections were prepared using a Microm Cool-Cut microtome at room temperature. Tissue sections were placed on 1 mm glass slides coated with a 1.35 µm polyethylene naphthalene membrane (PALM). Deparaffinization was accomplished by three rinses in xylenes, then rehydrated with a graded series of ethanol washes (100–70%), followed by rinsing in deionized water. Tissue sections were stained with Dako Methyl Green (Dakocytomation) for 20 sec, rinsed with deionized water, then dehydrated with a graded series (70–100%) of ethanol washes and dried for 30 min at 37°C. A hematoxylin and eosin-stained tissue section was prepared for each tissue specimen for pathologic review and to guide microdissection. Methodology for the isolation of cells from frozen tissue specimens and RNA preparation is described below under “Gene Expression Profiling”.

### DNA Sequence and Allelotype Analyses

Sequence analysis of the *TP53* gene was accomplished as previously described in detail [44]. Exons 2–11 and flanking exon-intron boundaries were analyzed by direct sequence analysis for all samples. Genotyping and LOH analyses related to the *BRCA* founder mutations 185delAG, 5382insC (for *BRCA1*) and 6174delT (for *BRCA2*) were accomplished as previously described [45]. As all tissue specimens analyzed were from individuals harboring frameshift mutations, the mutant and wild-type alleles could readily be distinguished; loss of the wild-type allele was designated when the radiographic intensity of this allele was ≤25% of the mutant allele.

### Gene Expression Profiling

Normal ovarian tissues and ovarian carcinoma specimens were flash frozen at the time of surgery, embedded in O.C.T. Compound (Tissue-Tek), and stored at −80°C. A hematoxylin and eosin-stained tissue section was prepared from each tissue specimen and examined microscopically for evidence of epithelial cystic inclusions and intact surface epithelium. Seven such cases were judged to have sufficient cystic and surface epithelium for molecular analyses. Ovarian cancer specimens were subjected to a similar histopathologic review, and 11 cases of advanced stage, serous ovarian carcinoma were selected for molecular analyses. For laser catapult microdissection, 8 µm sections from the corresponding frozen, O.C.T.-embedded specimens were prepared using a Thermo Cryotome at −20°C, then placed on 1 mm glass slides coated with a 1.35 µm polyethylene naphthalene membrane (PALM). Tissue sections were stained with Dako Methyl Green (Dakocytomation) for 2–3 min, rinsed with deionized water, dehydrated and fixed with a graded series (70–100%) of ethanol washes, and dried in a vacuum-dessicator at room temperature for 20–30 min. Tissue sections were then immediately subjected to laser catapult microdissection.

From the total pool of normal ovarian tissues, approximately 10,000 cells were captured from cystic inclusions and 10,000 cells were captured from the surface epithelium; a similar number of cells was obtained from the ovarian carcinomas. Laser catapult microdissection was accomplished using the PALM MicroBeam System (as above). After catapulting the cells of interest, each group of cells (normal ovarian cyst epithelium, normal ovarian surface epithelium, and ovarian carcinoma) was divided into three samples each, for a total of nine cell samples. Digestion of cells was accomplished as described above, followed by total RNA isolation using the Absolutely RNA Nanoprep Kit (Stratagene). Reverse transcription was performed using oligo(dT) primers and the SuperScript Double-Stranded cDNA Synthesis kit (Invitrogen). Synthesis and linear amplification of cRNA were accomplished by transcription *in vitro* using the MessageAmp RNA kit (Ambion). Two rounds of linear amplification were performed on all nine samples. Labeling of cRNA was performed during a third round of amplification using biotinylated nucleotides (Enzo Diagnostics). The length of labeled cRNA was assessed using the Agilent Bioanalyzer 2100 nanoAssay; the average length was 600 nt. Labeled cRNA samples were then hybridized to the Human Genome U133A GeneChip (Affymetrix), containing 22,215 oligonucleotide-based probe sets, at 45°C for 16 hr. Automated washing and staining were performed using the Affymetrix Fluidics Station 400 according to the manufacturer's protocols, and probe intensities were quantified using the argon laser confocal GeneArray Scanner (Hewlett-Packard).

Raw expression data were normalized to a target intensity of 500 to account for differences in global chip intensity, and expression values were then transformed using the logarithm base 2. Probe sets with very low average expression were eliminated because their expression measurements were not reliable. A threshold of 6 on the log scale was used for this purpose. Differences in absolute gene expression levels between cyst and surface, tumor and surface, and tumor and cyst were determined using a two-sample *t*-test. Results for cyst compared to surface (Supplemental Table S4), cyst and tumor compared to surface (Supplemental Table S5), and surface and tumor compared to cyst (Supplemental Table S6) are presented. All genes with *P*-values less than 0.05 are listed. In the latter two tables, genes were listed when both *P*-values were less than 0.05 and the fold-changes were in the same direction.

### DNA Content Quantitation

The same formalin-fixed, paraffin-embedded ovarian tissue specimens used for quantitation of the proliferation index (see Results section) were used to determine DNA content. These were unselected, pathologically normal ovaries (*n* = 36) removed from women for benign indications who were not at increased genetic risk for cancer. To avoid sectioning through nuclei, 10 µm sections were prepared and placed on glass slides as described above for immunohistochemical analyses. The human breast cancer cell lines MCF7 and SK-BR-3 were obtained from the American Type Culture Collection. These cell lines, known to be hypertriploid, were pelleted, fixed and embedded, and processed as above to served as positive cellular controls for aneuploidy. Slides were deparaffinized and rehydrated as for immunohistochemistry, stained with propidium iodide at 10 µg/ml in the presence of RNase A, then stored in PBS until DNA quantitation.

Quantitation of DNA content was accomplished using the ApoTome Imaging System (Carl Zeiss). Slides were viewed on the Axioplan 2 imaging microscope at 400X magnification, and images were captured through the AxioCam MR monochrome digital camera providing a resolution of 1300×1300 pixels. The AxioVision 4.1 image acquisition software was used to obtain optical sections at 0.85 µm intervals through selected nuclei. Monochrome images were analyzed using the metaMorph Imaging System (Universal Imaging Corporation). Nuclei were outlined manually with region tracing tools and an integrated intensity over the total area of each nucleus was calculated. Each pixel was assigned an intensity value from 0 (pure black) to 255 (pure white). Overlapping nuclei were excluded from the analysis.

### Ploidy Quantitation by Fluorescence in situ Hybridization

The same ovarian tissue specimens used for determination of proliferation index and DNA content were used for ploidy analysis. Sections of 4 µm thickness were placed on glass slides, deparaffinized, and rehydrated as above, then pretreated with sodium thiocyanate and digested with proteinase according to the probe manufacturer's protocol (Vysis). Tissue sections were then subjected to denaturation at 73°C for 5 min followed by dehydration in a graded series of ethanol washes (70–100%). Six chromosome enumeration probes (Vysis), directed toward the centromeric alpha satellite regions of chromosomes 1, 3, 6, 7, 8 and 11, were labeled with an appropriate fluorophore and hybridized to tissue specimens using a codenaturation protocol provided by the manufacturer. Two to three probes were generally applied together. Slides were coverslipped and incubated at 73°C for 5 min followed by 42°C for 16 hr, then washed and counterstained with DAPI. Fluorescence quantitation was accomplished using the same technology described above for DNA quantitation, except that slides were examined at 1000X using triple or quadruple fluorescence, and each fluorophore was exposed individually through the appropriate filter. Image overlays were reconstructed to determine the relationship between specific nuclei and probes. Only probe signals within nonoverlapping nuclei were enumerated and included in the analysis. The number of probes per chromosome per nuclei was recorded for both surface and cystic epithelial cells as described in the Results section. The chi-square test was used to assess statistical significance for the data presented in Table 3.

### Additional Statistical Analyses

The chi-square test was used to determine statistical significance of FOS and TOP2A expression differences between cyst and surface epithelium of the ovaries examined (Figure 3). Because of the paired, non-Gaussian nature of the data, a permutation t-test (100,000 permutations) was used to assess statistical independence of the cell proliferation (Ki-67) and apoptosis (TUNEL) variables between cyst and surface epithelial cell populations (Table 1). For quantitative comparisons of FOS, TOP2A, and Ki-67 expression, and of apoptosis between ovarian surface and cystic epithelium, all cells analyzed were classified as either positive or negative. The EASE (Expression Analysis Systematic Explorer) theme discovery tool [43] was used to identify genes comprising molecular biological pathways potentially overrepresented in microarray-generated gene expression data (Supplemental Table S7). This algorithm identifies Gene Ontology (GO) terms that describe a statistically significant number of genes in a list (Supplemental Table S5, in this case) compared to the population of genes from which the list was derived (those represented on the Affymetrix U133A microarray). A variation of the one-tailed Fisher exact probability test is used to generate an “EASE score” (*P*-value) for a given GO category over-represented on the gene list [43]. For DNA content quantitation, which involved the comparison of three groups, mean intensity values of nuclei were compared between groups using a one-way analysis of variance (ANOVA). The exact Mann-Whitney statistic was used to determine the significance of differences in the prevalence of aneuploidy between cyst and surface epithelium (Table 3); the *P*-value was based on 100,000 permutations since the data were paired.

## Supporting Information

Table S1 Genetic alterations in p53-immunopositive epithelial cells from ovaries removed prophylactically from BRCA heterozygotes.
(0.03 MB DOC)

Table S2 Genetic alterations in stage I ovarian carcinomas from BRCA heterozygotes.
(0.03 MB DOC)

Table S3 Mutation of the TP53 gene in progression of sporadic ovarian carcinoma.
(0.03 MB DOC)

Table S4 Genes differentially expressed in ovarian cystic epithelium compared to ovarian surface epithelium. “FC(C)” refers to the fold-change in ovarian cystic epithelium compared to ovarian surface epithelium, followed by the corresponding P-value.
(0.32 MB XLS)

Table S5 Genes differentially expressed in ovarian cystic epithelium and ovarian cancer compared to ovarian surface epithelium. “FC(C)” refers to the fold-change in ovarian cyst compared to ovarian surface epithelium, followed by the corresponding P-value; “FC(T)” refers to the fold-change in ovarian cancer compared to ovarian surface epithelium, followed by the corresponding P-value.
(0.17 MB XLS)

Table S6 Genes differentially expressed in ovarian surface epithelium and ovarian cancer compared to ovarian cystic epithleium. “FC(S)” refers to the fold-change in ovarian surface epithelium compared to ovarian cystic epithelium, followed by the corresponding P-value. “FC(T)” refers to the fold-change in ovarian cancer compared to ovarian cystic epithelium,
(0.08 MB XLS)

Table S7 Functional categories of genes differentially expressed in ovarian cystic epithelial and invasive cancer cells compared to surface epithelial cells. The gene category name is derived from Gene Ontology biological process or molecular function categories, and the P-value represents the “EASE score” as described in ref. 25.
(0.03 MB DOC)

Figure S1 BRCA1-linked ovarian carcinoma case OC7 (see Supplemental Table S2). The immunostain in all four panels is for p53. (A) Low-power photomicrograph displaying an inclusion cyst containing a transition of normal epithelium (N) to dysplasia (D) to invasive carcinoma (T). (B-D) High-power photomicrographs of the normal, dysplastic, and invasive cancer components, respectively, shown in panel (A), all strongly immunopositive for p53.
(10.07 MB TIF)

Figure S2 BRCA1-linked ovarian carcinoma case OC16 (see Supplemental Table S2). The immunostain in all four panels is for p53. (A) Low-power photomicrograph displaying an inclusion cyst containing a transition of normal epithelium (N) to dysplasia (D) to invasive carcinoma (T). (B-D) High-power photomicrographs of the normal, dysplastic, and invasive cancer components, respectively, shown in panel (A), all strongly immunopositive for p53.
(9.90 MB TIF)

Figure S3 Expression of p53 in unselected, pathologically normal ovary from patient not known to be at increased genetic risk for ovarian carcinoma. (A) Epithelium of inclusion cyst displaying strong p53 immunopositivity. (B) Surface epithelium from same ovary displaying undetectable levels of p53.
(5.09 MB TIF)

Figure S4 Sporadic ovarian carcinoma case S48 (see Supplemental Table S3). The immunostain in all four panels is for p53. (A) Low-power photomicrograph displaying an inclusion cyst containing a transition of normal epithelium (N) to dysplasia (D) to invasive carcinoma (T). (B-D) High-power photomicrographs of the normal, dysplastic, and invasive cancer components, respectively, shown in panel A, all strongly immunopositive for p53.
(0.86 MB TIF)

Figure S5 Sporadic ovarian carcinoma case S65 (see Supplemental Table S3). The immunostain in all four panels is for p53. (A) Low-power photomicrograph displaying an inclusion cyst containing a transition of normal epithelium (N) to dysplasia (D) to invasive carcinoma (T). (B-D) High-power photomicrographs of the normal, dysplastic, and invasive cancer components, respectively, shown in panel (A), all strongly immunopositive for p53.
(10.06 MB TIF)

Figure S6 Quantitation of cell proliferation indices in normal ovarian cystic and surface epithelia. (A,B) Quantitation of cell proliferation as determined by immunohistochemical assessment of Ki-67 expression in surface epithelial cells (A), and cystic epithelial cells (B), from the same ovary. Arrow in panel (B) indicates cystic epithelial cell immunopositive for Ki-67. (C,D) Quantitation of apoptosis as determined by TUNEL assay in surface epithelial cells (C), and cystic epithelial cells (D). Surface epithelial cells are strongly immunopositive and cystic epithelial cells are uniformly negative.
(8.90 MB TIF)

Figure S7 DNA content in cystic and surface epithelial cells from 36 normal ovaries. Surface epithelial cells (blue bars) are completely within the range of normal diploid control cells (not shown), whereas cystic epithelial cells (red bars) overlap substantially with aneuploid control cells (black bars).
(2.53 MB TIF)
