# Supplementary material for: Genetic Analysis of the Early Natural History of Epithelial Ovarian Carcinoma
Source: PLoS One. 2010 Apr 26;5(4):e10358. doi: 10.1371/journal.pone.0010358 (PMC2859950; doi:10.1371/journal.pone.0010358)
Supplement: Table S1 — Genetic alterations in p53-immunopositive epithelial cells from ovaries removed prophylactically from BRCA heterozygotes. (0.03 MB DOC) [file pone.0010358.s001.doc]

**Supplemental Table S1.** Genetic alterations in p53-immunopositive epithelial cells from

ovaries removed prophylactically from *BRCA* heterozygotes.

____________________________________________________________________________

Ovarian specimen *BRCA* mutation *BRCA* LOH* *TP53* mutation*

____________________________________________________________________________

PO3 *BRCA1* 4050del4 Yes H193R (CAT > CGT)

PO49 *BRCA1* 5382insC No H179R (CAT > CGT)

PO67 *BRCA2* 6174delT Yes S185N (AGC > AAC)

____________________________________________________________________________

The *BRCA* mutation status was obtained from patient records. *Loss of heterozygosity affecting

the wild-type *BRCA* allele, and *TP53* mutation status were determined in this study.
