# Supplementary material for: Genetic Analysis of the Early Natural History of Epithelial Ovarian Carcinoma
Source: PLoS One. 2010 Apr 26;5(4):e10358. doi: 10.1371/journal.pone.0010358 (PMC2859950; doi:10.1371/journal.pone.0010358)
Supplement: Table S2 — Genetic alterations in stage I ovarian carcinomas from BRCA heterozygotes. (0.03 MB DOC) [file pone.0010358.s002.doc]

**Supplemental Table S2.** Genetic alterations in stage I ovarian carcinomas from *BRCA* heterozygotes. ____________________________________________________________________________________________________ _____ Tumor Dysplasia Normal

Cancer Dysplasia Normal

Tumor Clinico- *BRCA1 TP53*  *BRCA*  *TP53 BRCA*  *TP53* *BRCA*  *TP53*

specimen pathologic mutation mutation LOH Mut IHC LOH Mut IHC LOH Mut IHC

_________________________________________________________________________________________________________

OC3 IA, E, 3 5382insC IVS8+1 (G>A) wt + - ND ND - - - -

OC6 IB, S, 2 185delAG H179R(CAT>CGT) wt + + wt + + wt - +

OC7 IC, S, 2 5382insC C275Y(TGT>TAT) wt + + - + + - + +

OC15 IC, C, 3 185delAG N239insT wt + - wt - - - - -

OC16 IC, E, 2 185delAG R282W(CGG>TGG) wt + + wt + + wt + +

_________________________________________________________________________________________________________

The clinicopathologic information includes surgical stage, histologic type (E, endometrioid; S, serous; C, clear cell) and FIGO grade. The *BRCA1* mutation status was obtained from patient records and the tumor *TP53* mutation status was determined in this study. Loss of heterozosity (LOH) affecting the wild-type *BRCA1* allele is indicated by “wt”, with (-) indicating no LOH. Presence of the relevant *TP53* mutation (mut) is indicated by (+), with (-) indicating absence of mutation. Presence of p53 nuclear immunopositivity, as determined by immunohistochemistry (IHC), is indicated by (+), with negative immunoreactivity indicated by (-).
