# Supplementary material for: Genetic Analysis of the Early Natural History of Epithelial Ovarian Carcinoma
Source: PLoS One. 2010 Apr 26;5(4):e10358. doi: 10.1371/journal.pone.0010358 (PMC2859950; doi:10.1371/journal.pone.0010358)
Supplement: Table S3 — Mutation of the TP53 gene in progression of sporadic ovarian carcinoma. (0.03 MB DOC) [file pone.0010358.s003.doc]

**Supplemental Table S3.** Mutation of the *TP53* gene in progression of sporadic ovarian carcinoma.

____________________________________________________________________________

Tumor Clinico- Cancer Dysplasia Normal

specimen pathologic Mut IHC Mut IHC Mut IHC

____________________________________________________________________________

S44 1C, S, 2 R342X - R342X - R342X -

S46 IIA, S, 3 R248Q + - + - +

S48 IA, S, 3 P142del3 + - + - +

S56 IIB, S, 3 S241delC + - + - +

S60 1A, C, 2 - - - - - -

S62 IIC, S, 3 Y327X + - + - +

S65 IIC, S, 3 C238Y + - + - +

S71 IC, C, 2 - - - - - -

S72 IIB, S, 2 - + - + - +

S73 IC, C, 2 - - - - - -

S86 IC, S, 3 - + - + - +

S87 IIC, S, 2 W146X + - + - +

S90 IIC, S, 3 R175H + - + - +

S91 IC, E, 1 - - - - - -

S101 IIC, S, 3 H233del25 - - - - -

S102 IC, E, 2 Y220C + Y220C + Y220C +

S103 IC, S, 2 - - - - - -

S107 IA, E, 1 - - - - - -

S113 1A, E, 2 - - - - - -

**Supplementary Table S3.** (cont.)

____________________________________________________________________________

Tumor Clinico- Cancer Dysplasia Normal

specimen pathologic Mut IHC Mut IHC Mut IHC

____________________________________________________________________________

S117 IIC, S, 2 - + - + - +

S120 IIC, S, 3 D259Y + - + - +

S138 1A, M, 1 - - - - - -

S141 1A , M, 2 - - - - - -

____________________________________________________________________________

The clinicopathologic information includes surgical stage, histologic type (E, endometrioid; S, serous; C, clear cell; M, mucinous) and FIGO grade. Mutation (Mut) of the *TP53* gene is specificed when present, with no mutation indicated by (-). Presence of p53 nuclear immunoreactivity, as determined by immunohistochemistry (IHC), is indicated by (+), with negative immunoreactivity indicated by (-).
