# Supplementary material for: Genetic Analysis of the Early Natural History of Epithelial Ovarian Carcinoma
Source: PLoS One. 2010 Apr 26;5(4):e10358. doi: 10.1371/journal.pone.0010358 (PMC2859950; doi:10.1371/journal.pone.0010358)
Supplement: Table S7 — Functional categories of genes differentially expressed in ovarian cystic epithelial and invasive cancer cells compared to surface epithelial cells. The gene category name is derived from Gene Ontology biological process or molecular function categories, and the P-value represents the “EASE score” as described in ref. 25. (0.03 MB DOC) [file pone.0010358.s007.doc]

**Supplemental Table S7.** Functional categories of genes differentially expressed in ovarian cystic epithelial and invasive cancer cells compared to surface epithelial cells. The gene category name is derived from Gene Ontology biological process or molecular function categories, and the *P*-value represents the “EASE score” as described in ref. 25.

______________________________________________________________________

Signal transducer activity (n = 45; *P* = 0.006)*

NRFA1 VIPR2 GHRHR RGS11 GPR44 CCL24 HPN

RGS2 WNT5A CRHR1 MTUS1 ITGB1 IRS3L C3

CRIM1 GABRB1 AKAP13 GRINL1A PDGFC RGS11 HFE

TOB1 GNB5 KLRC4 OPRS1 SCG3 SELPLG

KDR GABRA6 IL13RA1 OR1E1 PDGFD GRIN2A

NR4A2 BTN1A1 NRXN3 GDF3 RDH8 GABARAPL3

IL6ST BMP1 CXCR6 THRAP4 IL17B TNFRSF13B

Cell cycle (n = 27; *P* = 0.009)^†^

TOP2A BIRC5 AKAP8 DLEU1 MAPK1 BIN1 YEATS4

BUB3 ARHG RBBP6 NCOA6 SORT1 PMS2L8 REPIN1

MCM3 TFDP2 ESR1 CDK7 CCNE1 MCM5 TARDBP

RPA2 RFP2 BTG3 VEGF KRAS2 PMS2L2

Microtubule cytoskeleton organization and biogenesis (n = 5; *P* = 0.01)^†^

TBCC MAP7 PCNT2 TBCD PRC1

______________________________________________________________________

*All down-regulated in cyst epithelial and tumor cells compared to surface epithelial cells.

^†^All up-regulated in cyst epithelial and tumor cells compared to surface epithelial cells.
