# Supplementary figures and images for: Genetic Analysis of the Early Natural History of Epithelial Ovarian Carcinoma
Source: PLoS One. 2010 Apr 26;5(4):e10358. doi: 10.1371/journal.pone.0010358 (PMC2859950; doi:10.1371/journal.pone.0010358)

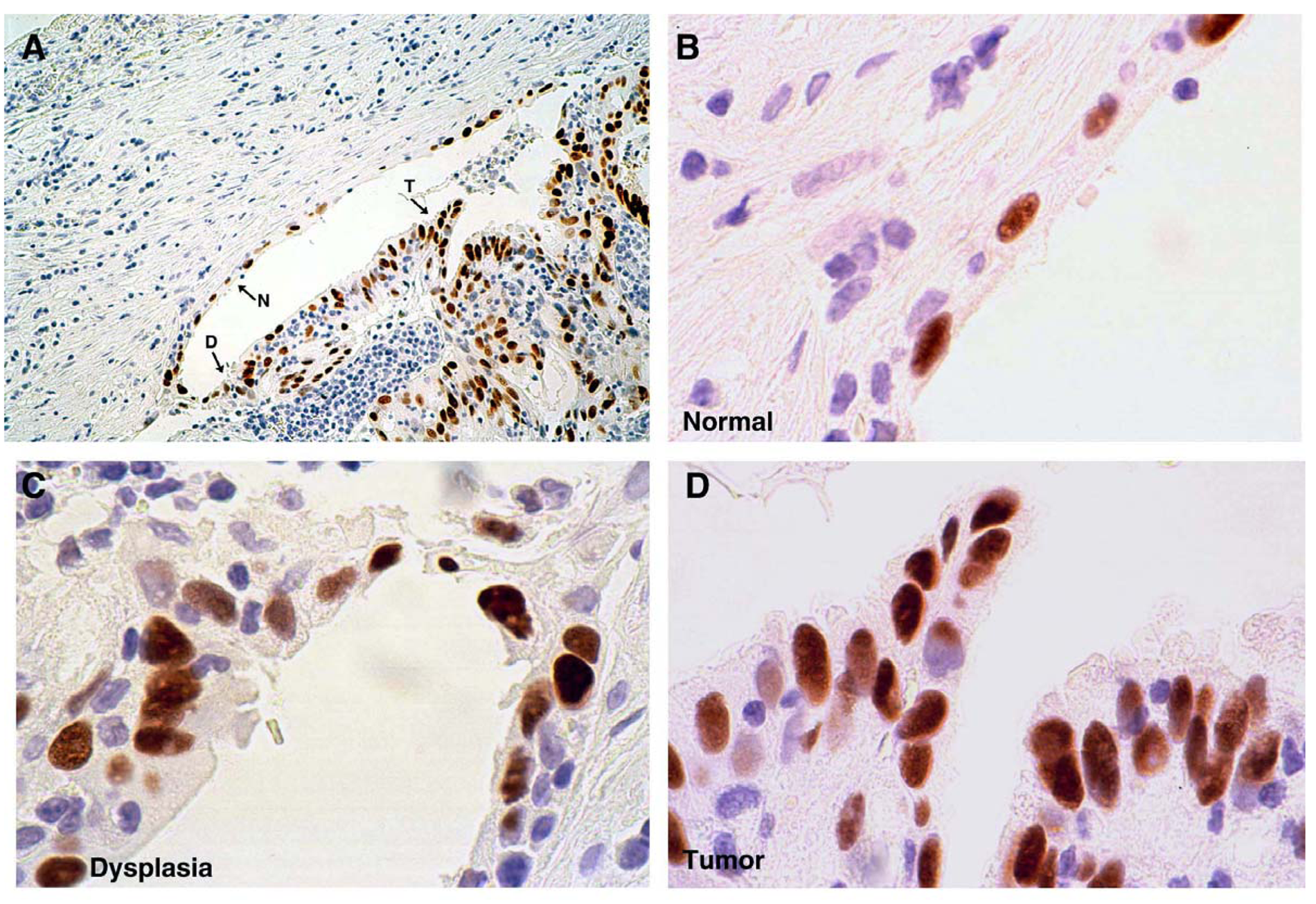

Supplement: Figure S1 — BRCA1-linked ovarian carcinoma case OC7 (see Supplemental Table S2). The immunostain in all four panels is for p53. (A) Low-power photomicrograph displaying an inclusion cyst containing a transition of normal epithelium (N) to dysplasia (D) to invasive carcinoma (T). (B-D) High-power photomicrographs of the normal, dysplastic, and invasive cancer components, respectively, shown in panel (A), all strongly immunopositive for p53. (10.07 MB TIF) [file pone.0010358.s008.tif]

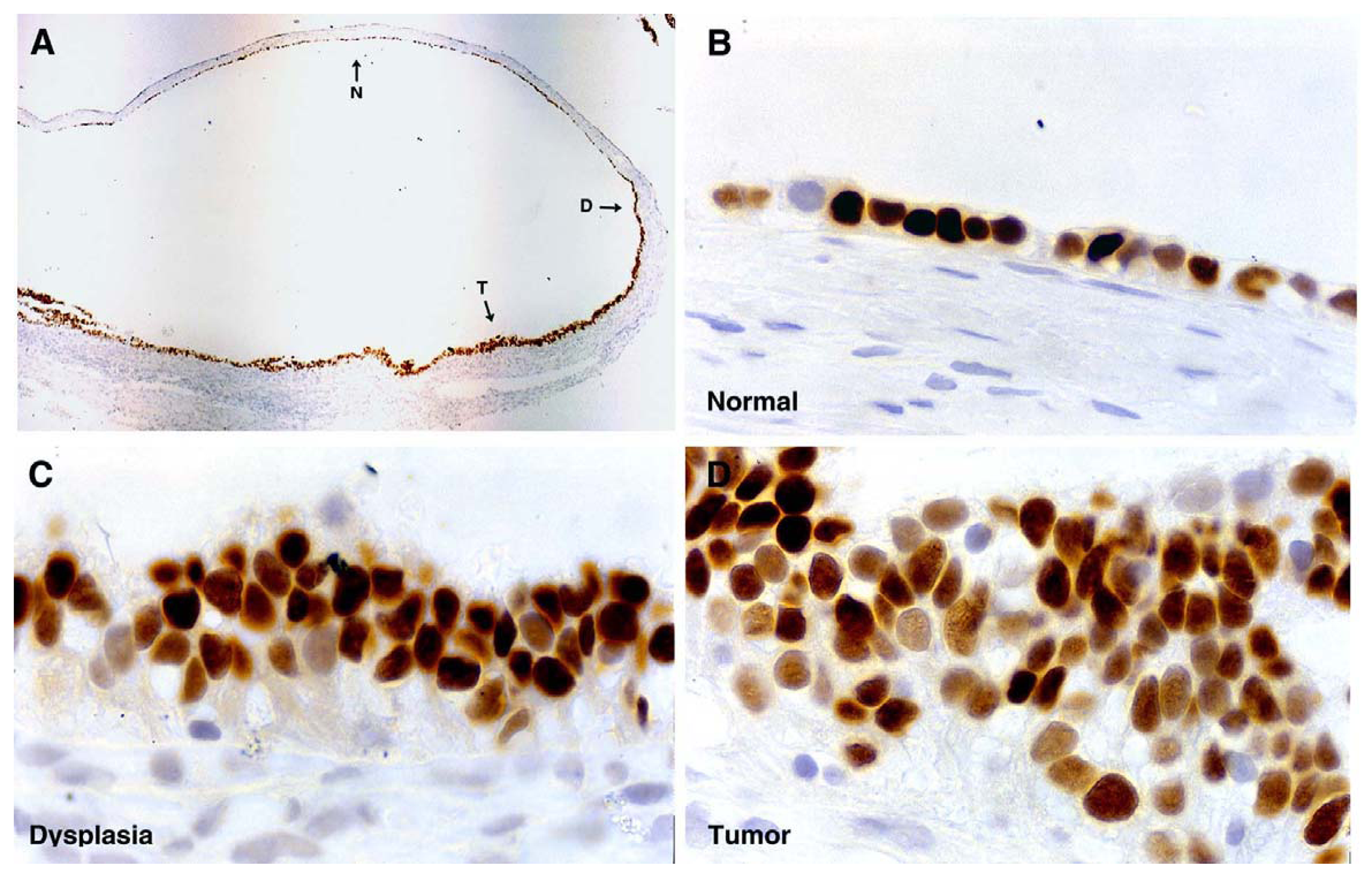

Supplement: Figure S2 — BRCA1-linked ovarian carcinoma case OC16 (see Supplemental Table S2). The immunostain in all four panels is for p53. (A) Low-power photomicrograph displaying an inclusion cyst containing a transition of normal epithelium (N) to dysplasia (D) to invasive carcinoma (T). (B-D) High-power photomicrographs of the normal, dysplastic, and invasive cancer components, respectively, shown in panel (A), all strongly immunopositive for p53. (9.90 MB TIF) [file pone.0010358.s009.tif]

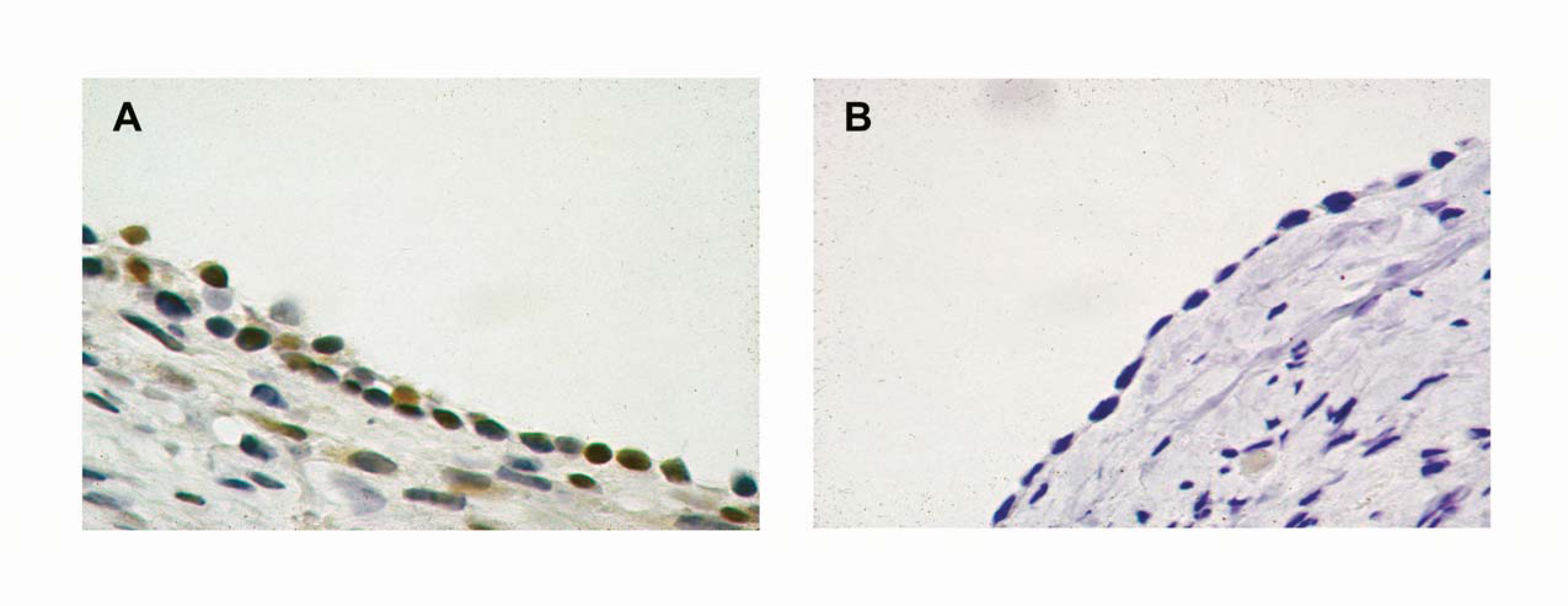

Supplement: Figure S3 — Expression of p53 in unselected, pathologically normal ovary from patient not known to be at increased genetic risk for ovarian carcinoma. (A) Epithelium of inclusion cyst displaying strong p53 immunopositivity. (B) Surface epithelium from same ovary displaying undetectable levels of p53. (5.09 MB TIF) [file pone.0010358.s010.tif]

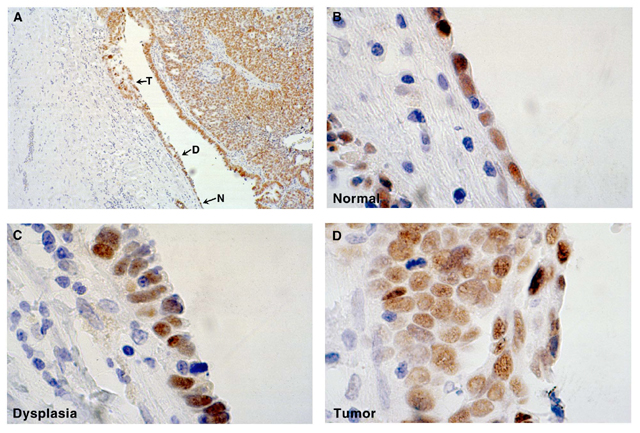

Supplement: Figure S4 — Sporadic ovarian carcinoma case S48 (see Supplemental Table S3). The immunostain in all four panels is for p53. (A) Low-power photomicrograph displaying an inclusion cyst containing a transition of normal epithelium (N) to dysplasia (D) to invasive carcinoma (T). (B-D) High-power photomicrographs of the normal, dysplastic, and invasive cancer components, respectively, shown in panel A, all strongly immunopositive for p53. (0.86 MB TIF) [file pone.0010358.s011.tif]

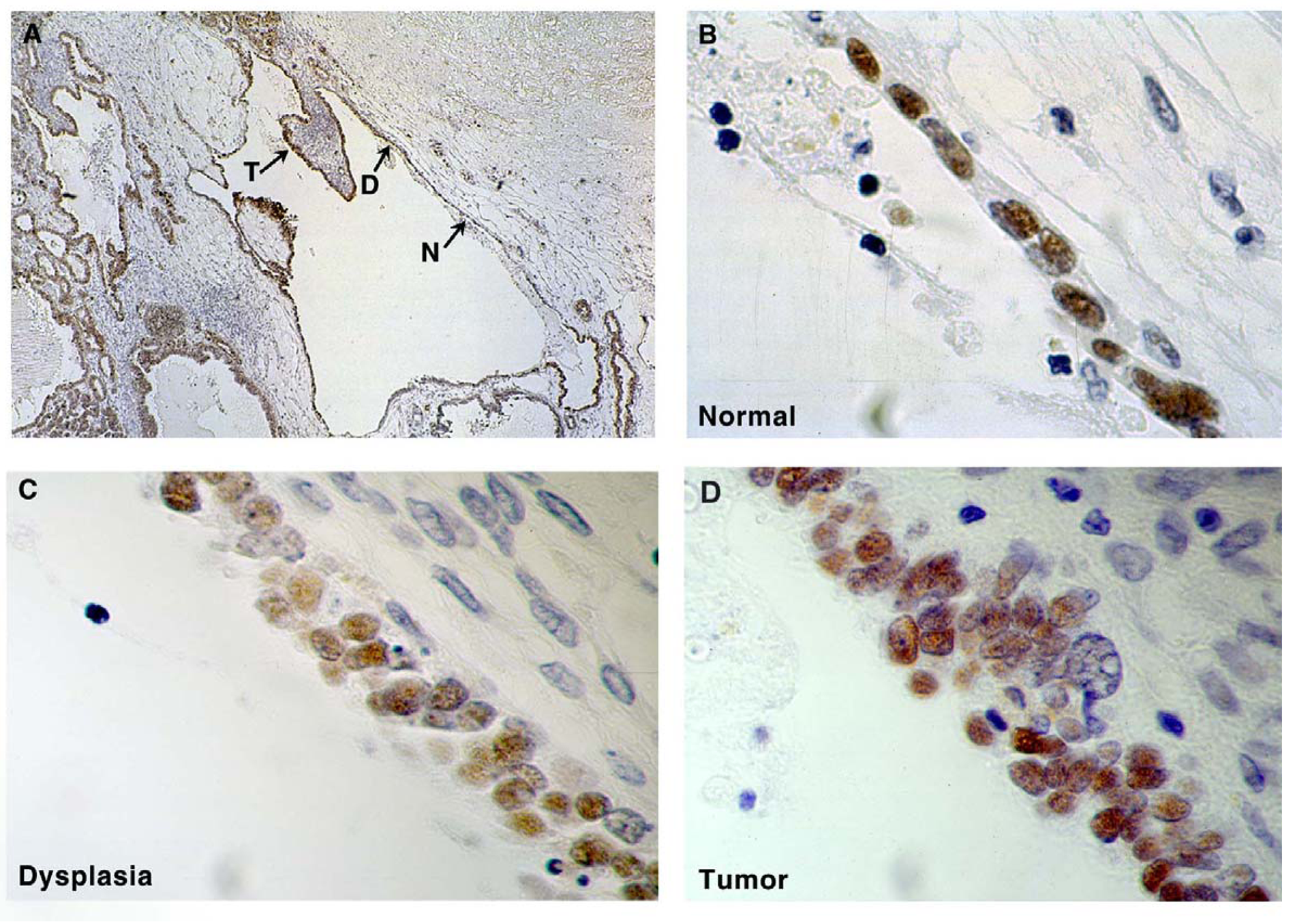

Supplement: Figure S5 — Sporadic ovarian carcinoma case S65 (see Supplemental Table S3). The immunostain in all four panels is for p53. (A) Low-power photomicrograph displaying an inclusion cyst containing a transition of normal epithelium (N) to dysplasia (D) to invasive carcinoma (T). (B-D) High-power photomicrographs of the normal, dysplastic, and invasive cancer components, respectively, shown in panel (A), all strongly immunopositive for p53. (10.06 MB TIF) [file pone.0010358.s012.tif]

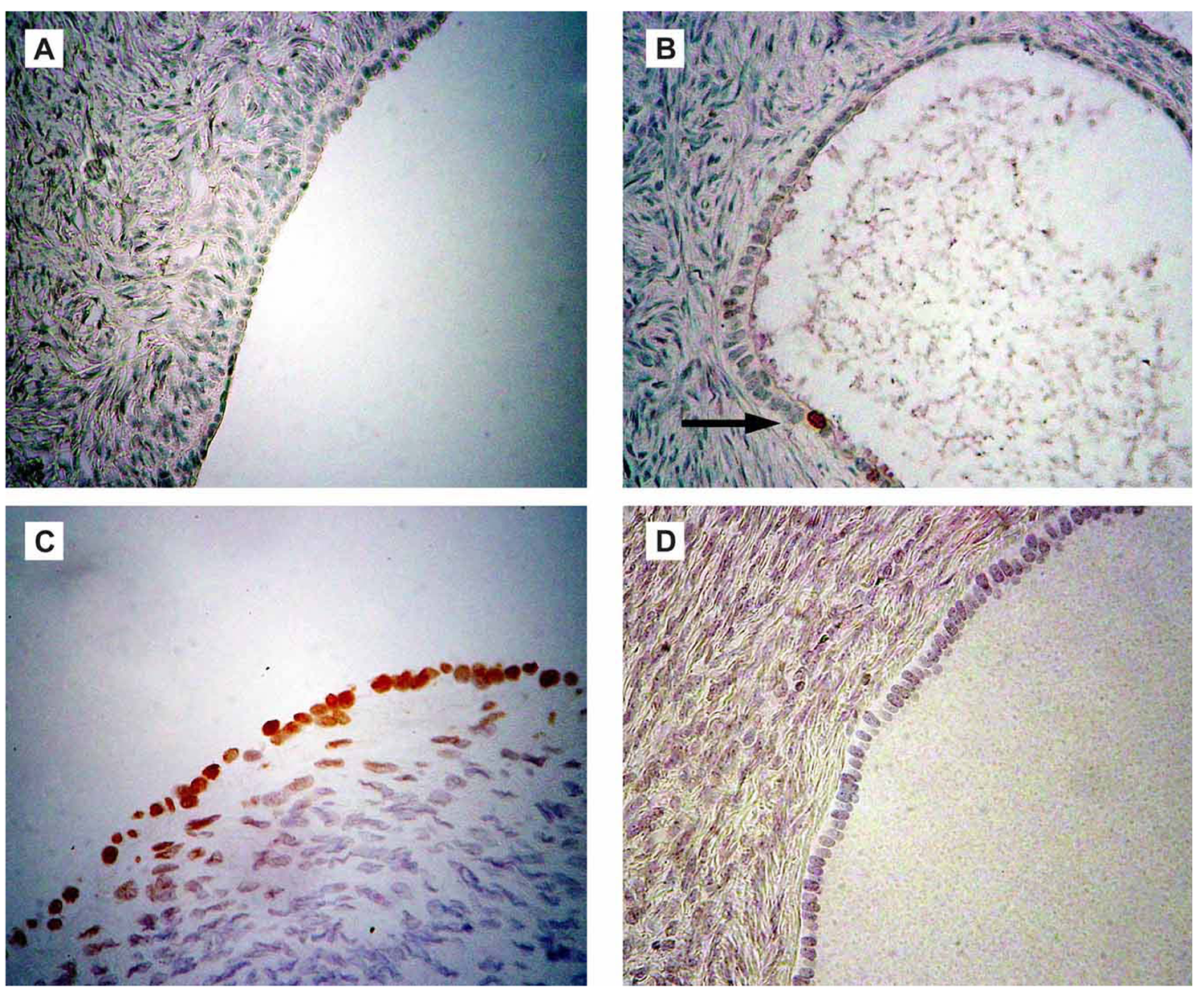

Supplement: Figure S6 — Quantitation of cell proliferation indices in normal ovarian cystic and surface epithelia. (A,B) Quantitation of cell proliferation as determined by immunohistochemical assessment of Ki-67 expression in surface epithelial cells (A), and cystic epithelial cells (B), from the same ovary. Arrow in panel (B) indicates cystic epithelial cell immunopositive for Ki-67. (C,D) Quantitation of apoptosis as determined by TUNEL assay in surface epithelial cells (C), and cystic epithelial cells (D). Surface epithelial cells are strongly immunopositive and cystic epithelial cells are uniformly negative. (8.90 MB TIF) [file pone.0010358.s013.tif]

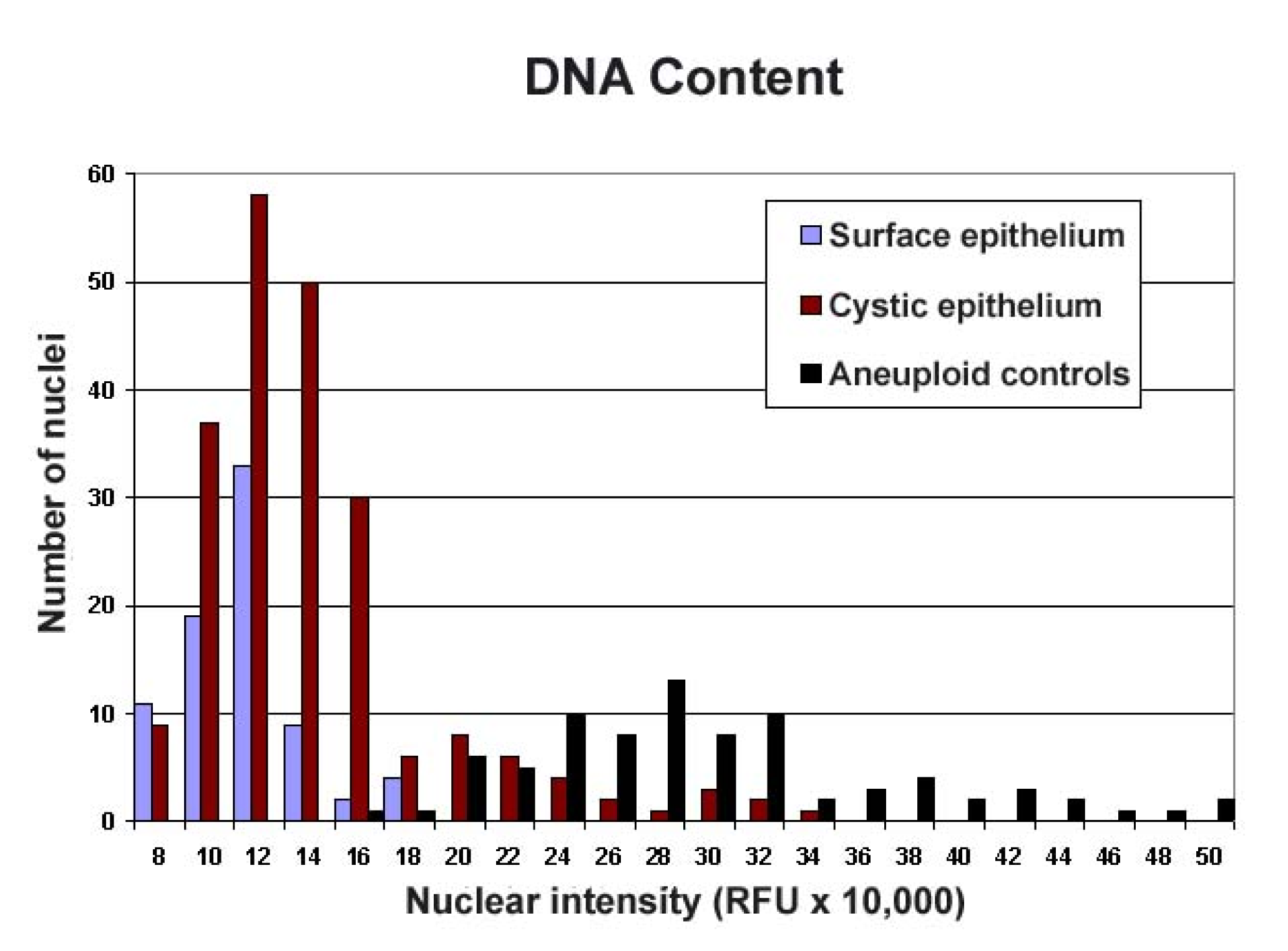

Supplement: Figure S7 — DNA content in cystic and surface epithelial cells from 36 normal ovaries. Surface epithelial cells (blue bars) are completely within the range of normal diploid control cells (not shown), whereas cystic epithelial cells (red bars) overlap substantially with aneuploid control cells (black bars). (2.53 MB TIF) [file pone.0010358.s014.tif]
